# Antioxidant efficacy of *Cotoneaster nummularius* in phenylhydrazine-induced hyperbilirubinemia: A rat model study

**DOI:** 10.22038/ajp.2025.26368

**Published:** 2026

**Authors:** Faezeh Valipour, Akbar Safipour Afshar, Mohammad Azadbakht, Hamidreza Mohammadi, Rahele Zhiani

**Affiliations:** 1 *Department of Biology, Neyshabur Branch, Islamic Azad University, Neyshabur, Iran*; 2 *Department of Pharmacognosy, Faculty of Pharmacy, Mazandaran University of Medical Sciences, Sari, Iran *; 3 *Medicinal Plants Research Center, Mazandaran University of Medical Sciences, Sari, Iran*; 4 *Department of Chemistry, Neyshabur Branch, Islamic Azad University, Neyshabur, Iran*

**Keywords:** Neonatal jaundice, Hyperbilirubinemia, Oxidative stress, Cotoneaster nummularius, Antioxidant defense

## Abstract

**Objective:**

This study investigated the hepatoprotective effects of *Cotoneaster nummularius* manna extract (CNE) against phenylhydrazine (PHZ)-induced hyperbilirubinemia and oxidative stress in a neonatal rat model.

**Materials and Methods:**

Fifty neonatal Wistar rats (2 weeks old) were divided into five groups (n=10): a control group, a PHZ-only group, and three PHZ-treated groups receiving CNE (1, 2.5, and 5 mg/kg, orally, thrice daily for 10 days). PHZ was used to induce hemolysis and hyperbilirubinemia. Markers of liver function, oxidative stress, and antioxidant capacity were analyzed, alongside β-glucuronidase activity.

**Results:**

CNE significantly mitigated PHZ-induced hyperbilirubinemia by reducing serum bilirubin levels and dose-dependently decreasing oxidative stress markers, including reactive oxygene species ROS, malondialdehyde (MDA), and protein carbonylation. It also restored glutathione (GSH) levels and total antioxidant capacity. The highest CNE dose (5 mg/kg) demonstrated the most pronounced effects. Furthermore, CNE inhibited β-glucuronidase activity, contributing to its hepatoprotective action. Hierarchical clustering and heatmap analyses corroborated the dose-dependent antioxidant and hepatoprotective properties of CNE.

**Conclusion:**

These findings highlight the hepatoprotective potential of *C. nummularius* extract in reducing oxidative stress and hyperbilirubinemia. CNE dose-dependent effects, particularly at 5 mg/kg, suggest its promise as a therapeutic agent for neonatal liver dysfunction and oxidative damage. Further clinical studies are warranted to explore its potential applications in managing liver disorders.

## Introduction

Bilirubin, the catabolic byproduct of heme metabolism, is a compound of significant clinical interest due to its dual role as both a potential neurotoxin and an antioxidant (Zhang et al. 2023). In neonates, the condition known as hyperbilirubinemia is characterized by elevated serum levels of unconjugated bilirubin, which may precipitate jaundice (Mitra and Rennie 2017). The liver, the central organ in bilirubin clearance, orchestrates its detoxification and subsequent excretion, thereby preventing its systemic accumulation (Yeudall et al. 2024). Dysfunctions in hepatic bilirubin processing pathways can precipitate hyperbilirubinemia, posing risks of neurological damage and increased mortality in the neonatal population (Huang et al. 2019).

The prevalence of neonatal jaundice poses a significant clinical challenge, with the potential for severe neurological outcomes including kernicterus, in otherwise healthy full-term infants (Yeudall et al. 2024). The pathogenesis of hyperbilirubinemia is multifactorial, encompassing increased bilirubin production, impaired conjugation, diminished hepatic uptake, and augmented bilirubin enterohepatic circulation (Thakur et al. 2024). Globally, hyperbilirubinemia stands as a critical factor in neonatal morbidity, rehospitalization, and mortality (Li et al. 2024). The primary intervention, phototherapy, utilizes blue spectrum light to facilitate bilirubin degradation, thereby reducing serum levels (Li et al. 2024). Exchange transfusion is reserved for severe cases unresponsive to phototherapy (Pathak et al. 2022). The efficacy of phototherapy is contingent upon the light’s wavelength, intensity, the extent of body surface area exposed, and the duration of treatment.

In recent decades, the exploration of medicinal plants for their traditional uses has garnered increasing scientific interest (Cervello et al. 2024). The quest for natural hepatoprotective agents from the plant kingdom, particularly those devoid of adverse effects, has intensified (Ali et al. 2019). Among the diverse flora, *Cotoneaster nummularius*, a Rosaceae family shrub, has been traditionally employed in managing neonatal jaundice (Nadaf et al. 2023). *Cotoneaster* species are valuable sources of a substance known as manna, a viscous juice synthesized by young shoots in response to damage inflicted by parasitic insects (Kicel 2020). Manna contains high concentrations of polysaccharide fraction with predominantly mannitol and a diverse array of phenolic and flavonoid compounds, contributing to its pharmacological potency. Recent studies have reported different activities associated with manna derived from *Cotoneaster* species, including antioxidant, anti-inflammatory, antibacterial, anticancer, and hepatoprotective activities (Kicel 2020). In Iranian folk medicine, the administration of *C. nummularius* extract to breastfed infants is a common practice for jaundice mitigation (Kicel 2020; Nadaf et al. 2023). Despite widespread traditional usage, a rigorous scientific evaluation of *C. nummularius* in clinically relevant neonatal hyperbilirubinemia models is lacking. 

Therefore, the current study utilizes a neonatal rat model of phenylhydrazine (PHZ)-induced hyperbilirubinemia. PHZ is a well-established chemical inducer of hemolytic anemia and hyperbilirubinemia (Nawaz et al. 2016). Its mechanism involves inducing significant oxidative stress, which directly damages erythrocyte membranes, leading to hemolysis and a subsequent surge in bilirubin production from heme catabolism. This rapid increase in bilirubin overwhelms the neonatal liver's conjugating and excretory capacity, resulting in hyperbilirubinemia (Wang et al. 2021). Furthermore, the oxidative stress generated by PHZ directly targets hepatocytes, causing liver damage characterized by lipid peroxidation and protein oxidation, impairing hepatic function including bilirubin processing (Shirani et al. 2023). This model, therefore, reflects key aspects of clinical conditions involving hemolysis and oxidative liver stress leading to jaundice. The logical cascade is thus: PHZ induces systemic oxidative stress, causing both hemolysis (leading to increased bilirubin load) and direct hepatotoxicity (impairing bilirubin clearance), culminating in hyperbilirubinemia and liver damage. 

This research aims to systematically evaluate the antioxidant and hepatoprotective effects of *C. nummularius* extract (CNE) against this PHZ-induced pathology, thus bridging traditional knowledge and modern biochemical understanding for improved neonatal care.

## Materials and Methods

### Plant material collection and extraction methodology

Manna samples from *C. nummularius*, collected across Khorasan Razavi, Iran, were identified by a qualified botanist. A voucher specimen of the *C. nummularius* plant, from which the manna was collected, was prepared and deposited in the Herbarium of Mazandaran University's Pharmacognosy Department, with the assigned herbarium number 20014. Only the manna (exudate) of the plant was used in this study. A 100 g manna sample was dissolved in 100 ml distilled water and filtered using Whatman No. 1 filter paper. The resulting solution (CNE) was stored at -20°C for future analysis. Although the preparation involved dissolving *C. nummularius* manna in water without organic solvents, the resultant solution is referred to as an ‘extract’ in alignment with traditional and pharmacognostic naming conventions that classify aqueous solutions of plant exudates as extracts.

### Animal selection and group allocation

In this study, we utilized fifty male neonatal Wistar rats, each precisely two weeks of age and within a weight spectrum of 25-30 g. The rats were procured from the controlled breeding facilities of Mazandaran University of Medical Sciences (MazUMS), Sari, Iran. The research protocol received full endorsement from the Institutional Animal Ethics Committee at MazUMS, ensuring adherence to ethical standards. The approval number for this study is IR.MAZUMS.REC.1399.420. Rigorous ethical guidelines were followed, with stringent measures implemented to minimize any form of discomfort, distress, or pain to the animals throughout the duration of the experimental procedures.

### Experimental design

Neonatal rats were housed under a 12-hr light/dark cycle with free access to maternal milk and water. Following 10 days of acclimatization, they were divided into five groups of 10. One control group received only the vehicle (1 ml/kg distilled water), while four experimental groups received oral doses of *C. nummularius* extract (CNE) at 0, 1, 2.5, and 5 mg/kg body weight, three times daily for 10 days. The 0 mg/kg CNE group served as a treated control, receiving the same administration schedule as the experimental groups but without the active compound. The selected doses of CNE were based on prior preliminary toxicity studies that confirmed the absence of hepatotoxic effects at these dosages. Animals were closely monitored throughout the study period for any signs of adverse effects including changes in behavior, feeding patterns, and weight loss, to ensure the safety and tolerability of CNE administration. After 10 days, rats were anesthetized with ketamine (80 mg/kg) and xylazine (10 mg/kg). Blood samples were collected carefully via cardiac puncture, with a maximum volume of 2 ml per animal, ensuring that the volume did not exceed approximately 7% of the total circulating blood volume, thus adhering to standard ethical guidelines for blood sampling in neonatal rodents. The livers were excised, placed on pre-cooled glass, weighed, and homogenized in 0.90% saline to create a 10% (w/v) homogenate using a sonicator. The homogenate was centrifuged at 3000×g for 20 min at 4°C, and the supernatant was collected for further biochemical assays.

### Induction of hyperbilirubinemia

In this study, hyperbilirubinemia was induced in 50 male neonatal Wistar rats, two weeks old. The rats were divided into two groups: a PHZ-treated group and a control group. Forty rats in the PHZ group received intraperitoneal injections of PHZ at a total dose of 70 mg/kg, administered in two equal doses 12 hours apart, while ten control rats received normal saline. Blood was collected 24 hr after the second PHZ injection for bilirubin analysis.

### Quantification of serum bilirubin levels

To assay the serum bilirubin concentration, blood specimens were initially centrifuged at 2000×g for 1 min to achieve serum separation. The isolated serum was then meticulously preserved at a temperature of −70°C to maintain its biochemical integrity until the analytical phase. The quantification of total serum bilirubin was executed employing the modified Jendrassik and Grof method, renowned for its precision in bilirubin measurement (Jendrassik and Grof 1938).

### Assessment of mitochondrial functionality

Mitochondrial functionality was evaluated using hepatic mitochondria isolated from male Wistar rats. Tissues were homogenized in ice-cold isolation buffer (250 mM sucrose, 10 mM Tris-HCl, 0.5 mM Ethylenediaminetetraacetic acid, dipotassium salt (EDTA-K+), 0.1% Bovine Serum Albumin (BSA), pH 7.4) and centrifuged at 2000 × g for 10 min at 4°C to remove debris. A second centrifugation at 10,000 × g for 10 min isolated mitochondria, resuspended in Tris-HCl buffer (0.25 M sucrose, 20 mM KCl, 2 mM MgCl_2_, 1 mM Na_2_HPO_4_, pH 7.4). Protein concentration was measured by the BCA assay. Functionality was assessed using the MTT assay, measuring absorbance at 570 nm after formazan formation (Arab-Nozari et al. 2020).

### Determination of mitochondrial reactive oxygen species (ROS)

Mitochondrial ROS levels were measured using the DCFH-DA assay, a fluorometric method. DCFH-DA, a non-fluorescent probe, is hydrolyzed by intracellular esterases to DCFH, which is oxidized by ROS to fluorescent DCF. The assay followed the manufacturer’s protocol (Novus Biologicals, USA). Fluorescence intensity of DCF was measured using a spectrophotometer set to an excitation wavelength of 500 nm and emission wavelength of 525 nm, providing a quantitative assessment of ROS activity.

### Analytical quantification of malondialdehyde (MDA) levels

Malondialdehyde (MDA) levels, a marker of oxidative stress, were quantified using the thiobarbituric acid reactive substances (TBARS) assay, as described by Boroushaki et al. (Boroushaki et al. 2014). Samples were standardized to 1 mg/ml protein using bovine serum albumin (BSA) as a reference. Then, 1 ml of homogenized sample was mixed with 2 ml of hydrochloric acid, 1 ml of 10% trichloroacetic acid, and 1.5 ml of 0.67% thiobarbituric acid. The mixture was incubated at 95°C for 30 min, cooled to room temperature, and centrifuged. The supernatant's absorbance, representing MDA concentration, was measured spectrophotometrically at 532 nm.

### Spectrophotometric analysis of protein carbonylation

Protein carbonylation was measured spectrophotometrically at 365 nm using 2,4-dinitrophenylhydrazine (DNPH) (Reznick and Packer 1994). Here, 100 mg of tissue was homogenized in 500 µl of 20% trichloroacetic acid (TCA) and incubated at 4°C for 15 min. Then, 500 µl of DNPH solution (10 mM in 2.5N HCl) was added to the sample precipitates, while controls received 2.5N HCl. Samples were incubated at 27°C for 1 hr, then precipitated with 500 µl of 20% TCA. After centrifugation, pellets were washed three times with a 1:1 ethanol:ethyl acetate mixture. Pellets were dissolved in 200 µl of 6M guanidine hydrochloride, and carbonyl content was determined from the differential absorbance between DNPH-treated and control samples.

### Quantitative evaluation of β-glucuronidase activity

The enzymatic activity of β-glucuronidase within hepatic tissues was quantified employing the Beta-Glucuronidase Activity Assay Kit (ZellBio GmbH, Ulm, Germany), meticulously following the manufacturer’s guidelines. The tissue samples were prepared in accordance with established laboratory protocols. The assay involved measuring the fluorescence intensity at a wavelength of 450 nm, with the samples incubated at 37°C for a duration of 20 min under conditions devoid of light. The enzymatic activity was then calculated on a per-unit tissue weight basis, ensuring a standardized measure of β-glucuronidase activity across all samples.

### Quantification of hepatic reduced glutathione (GSH)

Hepatic GSH levels were quantified using the ZellBio GSH Assay Kit (ZellBio GmbH, Germany) following the manufacturer’s protocol. This colorimetric method employs dithionitrobenzoic acid (DTNB), which reacts with GSH's sulfhydryl group to produce thio-nitrobenzoic acid, a vividly colored compound. The color intensity, directly proportional to GSH concentration, was measured spectrophotometrically at 420 nm, ensuring precise GSH quantification.

### Evaluation of antioxidant capacity via FRAP assay

The Ferric Reducing Antioxidant Power (FRAP) assay is predicated on the principle of the redox reaction, wherein ferric ion complexes (Fe^3+^), bound to 2,4,6-tri[2-pyridyl]-s-triazine (TPTZ), undergo reduction to the ferrous state (Fe^2+^) in the presence of electron-donating antioxidants from the test samples (Benzie and Strain 1997). This assay measures the conversion of the Fe³⁺-TPTZ complex to the blue-colored Fe²⁺-TPTZ complex as a direct indicator of antioxidant potency. The change in absorbance, indicative of the reduction process, is spectrophotometrically monitored at a wavelength of 593 nm, thereby quantifying the sample’s antioxidant capacity. 

### Statistical Analysis

Experimental data were analyzed using GraphPad Prism software, with results presented as mean ± SD. Statistical significance was assessed using one-way ANOVA followed by Tukey’s post hoc test (p<0.05). Multivariate analysis was performed using Python, and data clustering was visualized through a heat map generated via the CIMMiner platform (https://discover.nci.nih.gov/cimminer/home.do).

## Results

### Impact of CNE on serum bilirubin levels

The influence of CNE on serum bilirubin concentrations was investigated in rats. Baseline serum bilirubin levels were quantified at 0.6 ± 0.08 mg/dl in the control cohort. In contrast, a marked elevation in serum bilirubin was noted in the PHZ-administered group when juxtaposed with the control group, with the difference reaching statistical significance (p<0.01). Furthermore, administration of CNE at varying dosages (1, 2.5, and 5 mg/kg body weight) resulted in a notable dose-responsive decrement in serum bilirubin levels, as detailed in Table 1. While all three doses of CNE significantly reduced serum bilirubin compared to the PHZ-only group, the highest dose (5 mg/kg) showed the most pronounced reduction and was significantly more effective than the 1 mg/kg dose (p<0.05).

**Table 1:** Effect of C. nummularius extract (CNE) on serum total bilirubin level in phenylhydrazine (PHZ)-treated rats.

Groups	Total Bilirubin (mg/dl)
**Control**	0.6 ± 0.8
**CNE 5**	3.9 ± 0.70 ∗∗
**CNE 2.5**	5.8 ± 0.60 ∗
**CNE 1**	6.3 ± 0.60
**PHZ**	7.1 ± 0.90

Data are presented as the mean ± SD, with n=10 animals per group. *p<0.05 and **p<0.01 compared to the PHZ group.

### Evaluation of liver mitochondrial function and ROS generation post-CNE administration

**Figure 1:**
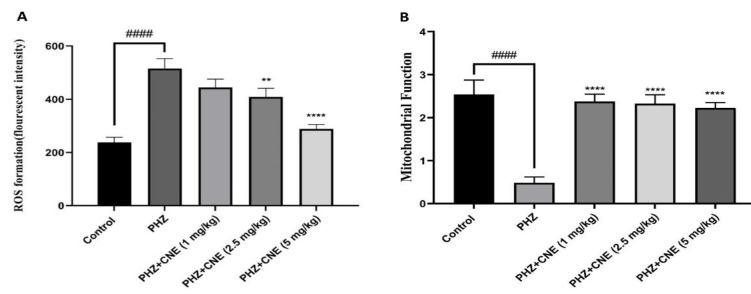
Effect of C. nummularius extract (CNE) on reactive oxygen species (ROS) formation of phenylhydrazine (PHZ)-induced liver toxicity in rats (A) and effect of treatments on mitochondrial function in isolated liver mitochondria (B). Data are presented as the mean ± SD, with n=10 animals per group. **###**p<0.001 compared to the control group; *p<0.05, **p<0.01 and ****p<0.0001 compared to the PHZ group.

Investigative results revealed a substantial augmentation in ROS production, exceeding twofold, in PHZ-treated rats compared to normative controls (p<0.05). Figure 1A illustrates that ROS accumulation was significantly mitigated upon treatment with CNE at dosages of 2.5 and 5 mg/kg body weight (p<0.05). A clear dose-dependent reduction in ROS was observed, with the 5 mg/kg dose resulting in significantly lower ROS levels compared to both the PHZ-only group (p<0.001) and the 1 mg/kg CNE dose group (p<0.05). The impact of CNE on liver mitochondrial function was quantified via the MTT assay, with Figure 1B demonstrating a significant enhancement in mitochondrial function following CNE treatment, relative to the control group (p<0.05). Mitochondrial function was significantly improved by all three doses of CNE (1, 2.5, and 5 mg/kg) compared to the PHZ-only group (p<0.05 for 1 mg/kg, p<0.01 for 2.5 mg/kg, and p<0.001 for 5 mg/kg). Although a trend towards dose-dependent improvement was observed, the differences in mitochondrial function among the CNE treatment groups did not reach statistical significance (p>0.05).

### Influence of CNE on antioxidant capacity (FRAP)

**Figure 2:**
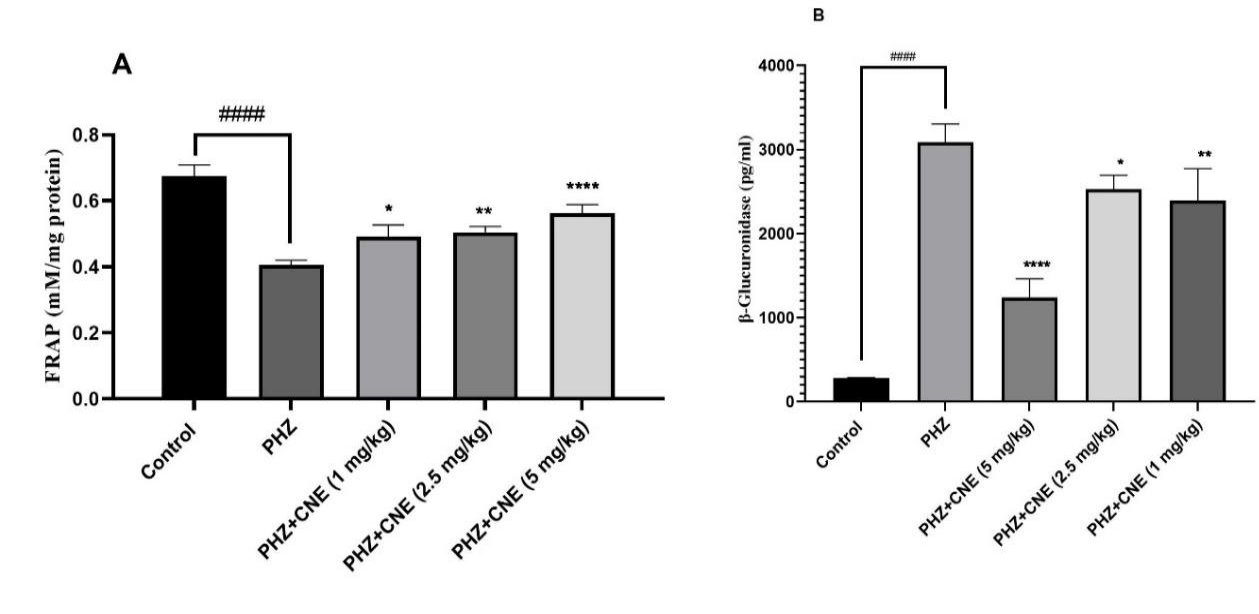
Effect of C. nummularius extract (CNE) on antioxidant capacity measured by ferric reducing antioxidant power (FRAP) assay (A) and β-glucuronidase activity (B) of phenylhydrazine (PHZ)-induced liver toxicity in Wistar rats. Data are presented as the mean ± SD, with n=10 animals per group. **###**p<0.001 and **####**p<0.0001 compared to the control group; *p<0.05, **p<0.01 and ****p<0.0001 compared to the PHZ group.

One-way ANOVA analysis followed by Tukey's multiple comparisons test revealed significant differences in antioxidant capacity among the treatment groups (p<0.05). PHZ treatment significantly reduced FRAP compared to the control group (p<0.0001). CNE administration at all doses (1, 2.5, and 5 mg/kg) significantly increased FRAP compared to the PHZ-only group (p<0.05), with the highest doses (2.5 and 5 mg/kg) showing the most pronounced effect. While all CNE doses significantly improved FRAP compared to the PHZ group, there was no significant difference between the 1 mg/kg and 2.5 mg/kg CNE groups (Figure 2A). However, the 5 mg/kg dose showed significantly higher FRAP levels compared to the 1 mg/kg dose (p<0.05). 

### Modulation of β-glucuronidase activity by CNE in PHZ-induced rats

The analysis revealed significant differences in β-glucuronidase activity among the treatment groups (p<0.05). PHZ treatment markedly increased β-glucuronidase activity compared to the control group (p<0.0001). As illustrated in Figure 2B, administration of CNE at doses of 1, 2.5, and 5 mg/kg significantly reduced β-glucuronidase activity relative to the PHZ-only group (p<0.05), with the 5 mg/kg dose exhibiting the most substantial effect (p<0.0001). Notably, while all CNE doses significantly decreased β-glucuronidase activity compared to the PHZ group, the 5 mg/kg dose was significantly more effective than both the 2.5 mg/kg and 1 mg/kg doses (p<0.0001). A clear dose-dependent reduction in β-glucuronidase activity was observed with increasing doses of CNE.

### CNE effect on GSH levels

**Figure 3:**
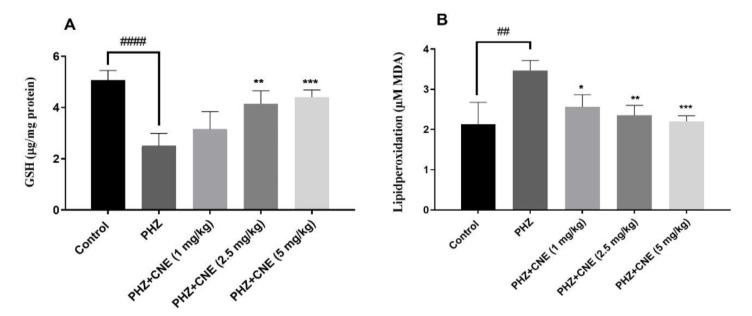
Effect of C. nummularius extract (CNE) on reduced glutathione (GSH) content (A) and lipid peroxidation (malondialdehyde (MDA)) (B) of phenylhydrazine (PHZ)-induced liver toxicity in Wistar rats. Data are presented as the mean ± SD, with n=10 animals per group. **##**p<0.01 and **####**p<0.0001 compared to the control group; **p<0.01 and ***p<0.001 compared to the PHZ group.

Figure 3A indicates a significant depletion of GSH content within the hepatic cells of PHZ-treated rats in comparison to controls (p<0.05). PHZ treatment significantly decreased GSH content compared to the control group (p<0.0001). CNE administration at 1 mg/kg significantly increased GSH content compared to the control group (p=0.0014), suggesting a protective effect against PHZ-induced GSH depletion. GSH levels at 2.5 mg/kg and 5 mg/kg CNE did not significantly differ from the control group; however, both doses significantly increased GSH content compared to the PHZ-only group (p=0.0027 and p=0.0008, respectively). Notably, CNE at 1 mg/kg significantly increased GSH content compared to both the 2.5 mg/kg and 5 mg/kg CNE groups (p=0.0224), indicating a potential dose-dependent response. Intriguingly, the 1 mg/kg dose appeared to be most effective in restoring GSH levels, showing a statistically significant difference compared to the higher doses.

### CNE’s role in attenuating lipid peroxidation

The influence of CNE on lipid peroxidation was assessed by measuring MDA levels, as shown in Figure 3B. PHZ administration significantly increased MDA levels compared to the control group (p=0.0011). However, CNE treatment at all tested doses (1, 2.5, and 5 mg/kg) did not significantly reduce MDA levels compared to the control group. Among the PHZ-treated groups, CNE administration at 1, 2.5, and 5 mg/kg significantly reduced MDA levels compared to the PHZ group, with the highest dose (5 mg/kg) showing the most substantial reduction (p=0.0010). A dose-dependent reduction in MDA levels was observed, with the 5 mg/kg dose demonstrating significantly greater efficacy in reducing MDA compared to the 1 mg/kg dose (p<0.05).

### Impact of CNE on protein carbonylation

**Figure 4:**
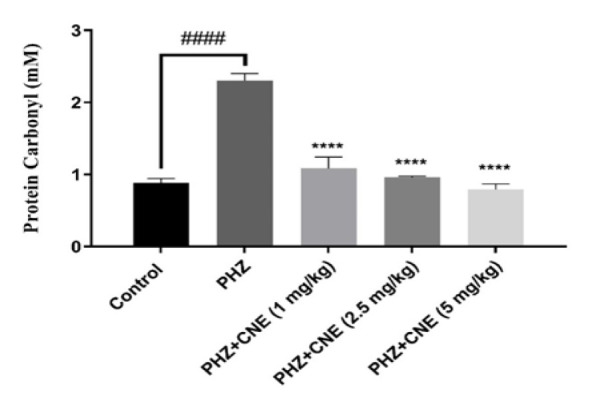
Protein carbonyl assay: Effect of C. nummularius extract (CNE) on the protein carbonylation of phenylhydrazine (PHZ)-induced liver toxicity in Wistar rats. Data are presented as the mean±SD, with n=10 animals per group. **####**p<0.0001 compared to the control group; ****p<0.0001 compared to the PHZ group.

Differential doses of CNE were assessed for their impact on protein carbonylation, a key marker of oxidative stress, as shown in Figure 4. PHZ treatment significantly increased protein carbonylation compared to controls (p<0.0001), indicating severe oxidative damage. CNE at 1, 2.5, and 5 mg/kg markedly reduced PHZ-induced protein carbonylation (p<0.0001 for all doses), with a dose-dependent effect observed. The 5 mg/kg dose was most effective, significantly outperforming the 1 mg/kg dose (p=0.0070). Importantly, protein carbonylation levels in all CNE-treated groups were comparable to controls (p>0.05), demonstrating CNE efficacy in restoring oxidative balance. A clear dose-dependent reduction in protein carbonylation was evident, with increasing doses of CNE leading to progressively lower levels, and the 5 mg/kg dose showing superior efficacy compared to both the 1 and 2.5 mg/kg doses (p<0.01 and p<0.05, respectively).

### Correlation analysis

**Figure 5:**
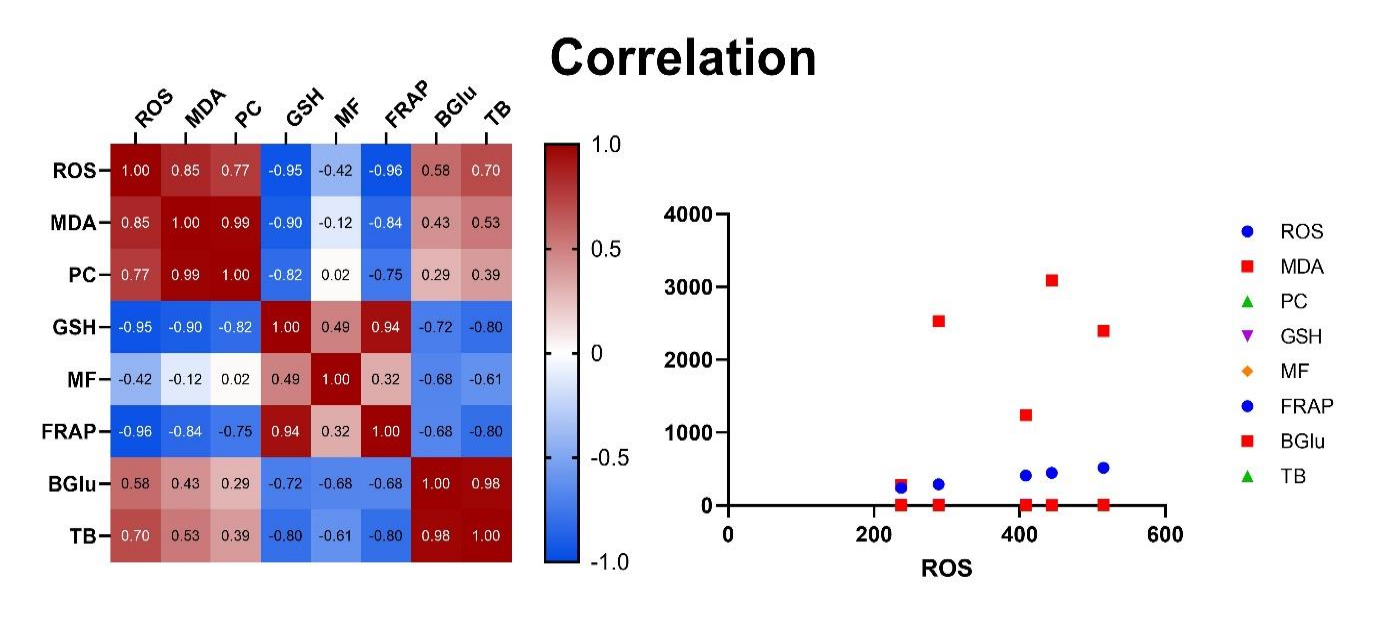
Correlation analysis illustrating the relationships between the biochemical parameters evaluated in the control and different treatment groups. This figure displays the correlation matrix and scatter plot, providing insights into the interdependencies of reactive oxygen species (ROS), malondialdehyde (MDA), protein carbonyl (PC), reduced glutathione (GSH), mitochondrial function (MF), ferric reducing antioxidant power (FRAP), β-glucuronidase activity (BGlu), and total bilirubin level (TB).

Correlation analysis, illustrated in the Figure 5, provides detailed insights into the interrelationships among the evaluated biochemical parameters. Strong positive correlations were observed between oxidative stress markers ROS and MDA (r=0.85, p<0.001) and between MDA and protein carbonylation (PC) (r=0.99, p<0.001), reflecting their concurrent increase under oxidative stress conditions induced by PHZ. Additionally, significant positive correlations were found between ROS and PC (r=0.77, p<0.001), emphasizing a coordinated oxidative response. 

Glutathione, mitochondrial functionality, and total antioxidant capacity demonstrated strong negative correlations with oxidative stress markers, notably ROS, MDA, and PC (r ranging from -0.75 to -0.90, p<0.001), confirming their antioxidative roles. Furthermore, β-glucuronidase (BGlu) activity displayed robust positive correlations with oxidative stress parameters (ROS, MDA, and PC) with correlation coefficients exceeding 0.70 (p<0.001), indicating its involvement in oxidative stress-induced hepatic dysfunction. Serum total bilirubin (TB) exhibited moderate to strong positive correlations with oxidative stress markers (r values from 0.53 to 0.61, p<0.01), highlighting the linkage between bilirubin accumulation and oxidative damage.

### Hierarchical clustering and heatmap analysis

**Figure 6:**
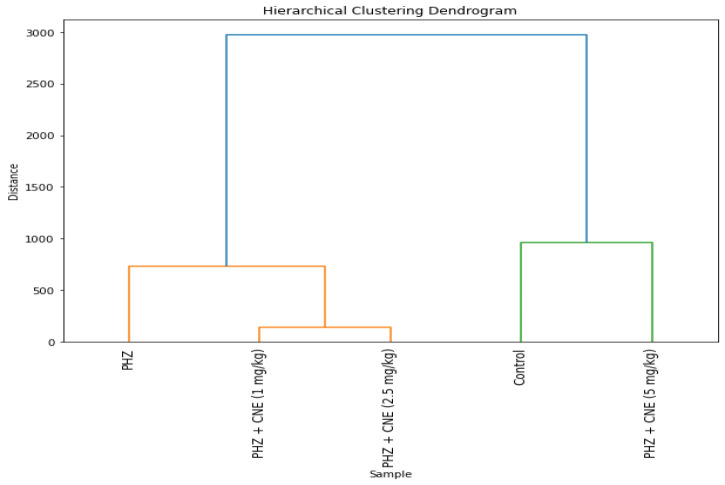
Hierarchical clustering dendrogram illustrating the pharmacological impact of varying doses of C. nummularius extract (CNE) on oxidative stress modulation in a phenylhydrazine (PHZ)-induced hyperbilirubinemia model. The dendrogram depicts two primary clusters: one encompassing the PHZ + CNE (1 mg/kg) and PHZ + CNE (2.5 mg/kg) treatment groups, denoting analogous modulatory effects, and another distinct cluster comprising the Control and the PHZ + CNE (5 mg/kg) group, reflecting a unique response to the higher CNE dosage. This visual representation underscores the dose-dependent pharmacological potential of CNE in oxidative stress management.

**Figure 7:**
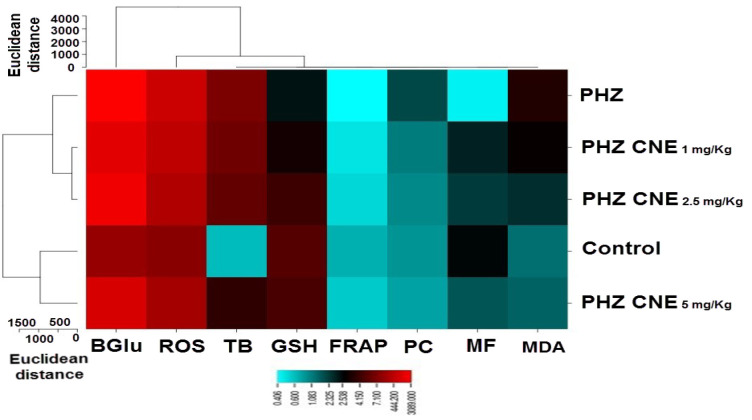
Heatmap illustrating the dose-responsive antioxidant effects of C. nummularius extract (CNE) in a phenylhydrazine (PHZ)-induced hyperbilirubinemia model in neonatal rats. The heatmap depicts the relative levels of reactive oxygen species (ROS), malondialdehyde (MDA), liver β-glucuronidase (BGlu) activity, and total antioxidant capacity (TP) across varying doses of CNE. A visible gradient from dark blue to dark red indicates the intensity of each parameter, with the highest dose of CNE (5 mg/kg) showing significant reductions in ROS, MDA, and BGlu, suggesting a potent antioxidative and hepatoprotective effect, while also enhancing the total antioxidant capacity as measured by the FRAP assay.

Hierarchical clustering analysis revealed distinct dose-dependent effects of CNE on oxidative stress biomarkers. The 1 and 2.5 mg/kg CNE groups exhibited similar biochemical responses, while the control and 5 mg/kg CNE groups formed a separate cluster, reflecting the stronger effects of the highest dose (Figure 6). Heatmap analysis confirmed dose-proportional antioxidative effects, with significant reductions in hepatic ROS and MDA levels. The 5 mg/kg dose notably decreased β-glucuronidase activity, highlighting its protective role against PHZ-induced hepatic dysfunction. Furthermore, CNE enhanced total antioxidant capacity, as shown by FRAP assay, particularly in the intermediate and high-dose groups (Figure 7).

## Discussion

The present study elucidates the remarkable antioxidative efficacy of CNE in mitigating oxidative stress and its associated consequences in a rodent model of PHZ-induced hyperbilirubinemia. Our findings demonstrate a dose-dependent reduction in serum bilirubin levels, ROS production, lipid peroxidation, and protein carbonylation, coupled with the restoration of depleted total antioxidant capacity following CNE administration. This research echoes and extends the findings of Fakhri et al. and Monsef et al., highlighting the reduction in serum bilirubin levels and the potential to curtail hospitalization durations (Fakhri et al. 2019; Monsef et al. 2019). Moreover, our study delineates the biochemical pathways modulated by CNE, particularly emphasizing the downregulation of oxidative stress markers such as MDA and protein carbonyls. The multifaceted mechanism of CNE action in neonatal hyperbilirubinemia involves both the mitigation of oxidative stress—evidenced by reduced MDA and protein carbonylation—and the bolstering of antioxidant defenses, as indicated by normalized GSH levels. These findings are in harmony with Aramipour et al., which corroborate the safety profile of CNE, thereby supporting its therapeutic potential (Aramipour et al. 2023). 

A particularly significant finding of this study is the clear dose-dependent efficacy of *C. nummularius* extract in ameliorating PHZ-induced hyperbilirubinemia and oxidative stress. Increasing doses of CNE, from 1 to 5 mg/kg, resulted in progressively greater reductions in serum bilirubin, hepatic ROS formation, MDA, and protein carbonylation. The highest dose of CNE (5 mg/kg) consistently demonstrated the most pronounced beneficial effects across multiple parameters, often showing statistically significant improvements compared to the lower doses. For instance, the 5 mg/kg dose was significantly more effective than the 1 mg/kg dose in reducing serum bilirubin, ROS, MDA, and protein carbonylation, and in increasing FRAP. This dose-response relationship provides strong evidence that the observed protective effects are directly attributable to the CNE treatment and are dependent on the administered dose, supporting a pharmacological action. Intriguingly, while higher doses were generally more effective in countering oxidative damage and reducing bilirubin, the 1 mg/kg dose of CNE appeared to be particularly effective in restoring hepatic GSH levels, showing a statistically significant increase compared to the higher doses. This suggests a potentially complex dose-dependent modulation of the antioxidant system, where lower doses might preferentially stimulate GSH synthesis or reduce its utilization, while higher doses exert broader antioxidant effects (Famurewa et al. 2023). This nuanced dose-response profile warrants further investigation to fully understand the optimal dosing strategies for different therapeutic goals. The clinical implications for neonatology are profound, suggesting CNE role as an adjunct to conventional phototherapy, offering a natural and minimally invasive alternative for managing neonatal jaundice. This is in line with the therapeutic versatility of CNE, as expounded by Sajedi and Fatollahierad, who underscored its utility in the management of neonatal hyperbilirubinemia (Sajedi and Fatollahierad 2019).

In synthesis, our study posits that the antioxidative properties of CNE hold significant promise for alleviating oxidative stress in neonatal hyperbilirubinemia. It beckons further inquiry to fully unveil its therapeutic potential and to devise standardized clinical application protocols. 

The current investigation utilized PHZ to induce neonatal hyperbilirubinemia, confirmed by elevated serum total bilirubin levels. PHZ operates through a dual mechanism relevant to this condition. Firstly, it induces potent oxidative stress, leading to the oxidation of erythrocyte components and subsequent hemolysis. This destruction of red blood cells drastically increases the amount of heme released, which is catabolized into bilirubin, thus significantly elevating the bilirubin load presented to the liver (Zhou et al. 2023). Secondly, the oxidative stress generated by PHZ directly damages liver cells, as evidenced in our study by increased hepatic ROS production, lipid peroxidation (MDA levels), and protein carbonylation in the PHZ-only group. This direct hepatotoxicity impairs the liver's capacity to effectively uptake, conjugate, and excrete the excess bilirubin, further contributing to the development of hyperbilirubinemia (Ahmed et al. 2024). Therefore, PHZ-induced hyperbilirubinemia is a consequence of both increased bilirubin production (due to hemolysis) and decreased hepatic clearance (due to liver damage), both stemming from PHZ-induced oxidative stress. Our findings demonstrate that CNE administration effectively counters these pathological processes. CNE mitigated oxidative stress markers (ROS, MDA, protein carbonylation), reduced bilirubin levels, inhibited β-glucuronidase activity (implicated in bilirubin deconjugation), and improved markers of antioxidant defense (GSH and FRAP), highlighting its potential to protect against both the hemolytic and hepatotoxic effects of PHZ (Perrone et al. 2023). The propensity of PHZ to amplify free radical production, induce lipid peroxidation, and cause erythrocyte lysis, results in hemolysis and subsequent hyperbilirubinemia (Memisoglu et al. 2017). Thus, countering oxidative stress presents a viable strategy against PHZ-induced hemolytic hyperbilirubinemia (Rajendran et al. 2024).

Our findings affirm PHZ potential to inflict damage on various cellular components, including lipids and proteins. This is supported by increased ROS production in hepatic tissue of PHZ-treated rats, leading to elevated MDA levels and protein carbonylation. The study observed a significant reduction in these elevated markers within the CNE-treated groups, suggesting CNE protective role against PHZ-induced toxicity and secondary hyperbilirubinemia. Furthermore, the study highlights the significant reduction in total antioxidant capacity and reduced GSH levels in the PHZ cohort, with CNE effectively reversing these effects. The antioxidative attributes of CNE mitigate the reduction in total antioxidant capacity and restore GSH levels, underscoring its potential as a protective food supplement or medicinal intervention for neonatal jaundice.

Correlation analysis revealed a strong positive correlation between the control group and the high-dose CNE group, indicating that CNE (5 mg/kg) effectively mitigates PHZ-induced biochemical alterations, aligning these parameters closer to the control. Strong positive correlations between the PHZ group and all CNE-treated groups, particularly at lower CNE doses, underscore the consistent efficacy of CNE in mitigating PHZ adverse effects. Furthermore, the strong negative correlation between the control and PHZ groups highlights the significant biochemical disruptions caused by PHZ, which were effectively countered by the high-dose CNE treatment. These findings reinforce the study's conclusion that CNE, particularly at higher doses, offers significant protection against PHZ-induced oxidative stress and liver damage.

The hierarchical clustering analysis substantiates the pivotal role of CNE dosage in modulating oxidative stress, delineating a dose-response relationship that is integral to the formulation of precise therapeutic strategies for neonatal health. This correlation necessitates further exploration into the specific biochemical markers influenced by varying CNE concentrations, which is essential for the development of evidence-based interventions in hyperbilirubinemia and associated neonatal disorders. The heatmap analysis further corroborates the capacity of CNE to modulate oxidative stress parameters in neonatal rats within a PHZ-induced hyperbilirubinemia framework. The observed dose-dependent diminution in ROS and MDA levels intimates the hepatoprotective potential of CNE, primarily through the attenuation of oxidative damage. The notable reduction in liver β-glucuronidase activity upon CNE administration underscores its efficacy in mitigating hepatic dysfunction. Moreover, the conservation of total antioxidant capacity and the augmentation of FRAP with CNE treatment suggest a contributory role in bolstering the hepatic antioxidant defense apparatus. 

Despite the promising antioxidant and hepatoprotective effects of CNE demonstrated in our neonatal rat model, several limitations should be acknowledged regarding its translation to human neonates. Differences in species-specific bilirubin metabolism, hepatic enzyme maturity, and antioxidant capacity could significantly influence the pharmacodynamics and efficacy of CNE in humans (Rajendran et al. 2024). Furthermore, the pharmacokinetic profile of CNE, including its absorption, distribution, metabolism, and excretion, remains unknown in neonatal humans and requires comprehensive evaluation. Although our dosing regimen showed safety in rats, extrapolation to clinical scenarios necessitates caution due to possible interspecies variability in metabolism and drug sensitivity. Current standard therapies for neonatal jaundice, particularly phototherapy and, in severe cases, exchange transfusion, are well-established, and any adjunctive therapy such as CNE must demonstrate clear additional benefits. The findings of this study suggest that CNE could enhance antioxidant defenses and possibly reduce the duration or intensity of phototherapy, but such hypotheses must be rigorously tested. Future research should prioritize detailed pharmacokinetic and toxicological studies in higher-order preclinical models, followed by carefully designed phase I/II clinical trials to establish safety, appropriate dosing, and preliminary efficacy in human neonates. These investigations should also evaluate CNE potential to work synergistically with phototherapy or pharmacologic agents like metalloporphyrins, offering a more comprehensive approach to managing neonatal hyperbilirubinemia.

In conclusion, our research underscores CNE capacity to alleviate the oxidative impairments of PHZ-induced hyperbilirubinemia in rat livers, with implications for oxidative stress mitigation. The study advocates for further exploration to ascertain the full scope of CNE applications in neonatal jaundice treatment.

## Data Availability

The data that support the findings of this study are available on request from the corresponding author.
